# Hyperglycosylated resurfaced stabilized GP120 core as an immunogen elicits antibodies targeted at the CD4-binding site

**DOI:** 10.1186/1742-4690-9-S2-P23

**Published:** 2012-09-13

**Authors:** K Dai, JC Boyington, W Shi, SD Schmidt, I Georgiev, D Lingwood, PD Kwong, JR Mascola, Z Yang, GJ Nabel

**Affiliations:** 1 Vaccine Research Center, NIAID, NIH, Bethesda, MD, USA

## Background

HIV-1 utilizes multiple mechanisms to evade immune surveillance. This has hindered the development of an effective vaccine. The CD4bs represents a highly conserved vulnerable site on HIV envelope that serves as an important target in HIV vaccine development. Novel concepts, such as resurfacing and glycan masking are some of the current approaches used to refocus the immune responses.

## Methods

In this study, we used structural information of HIV gp120 core, together with the computer-assisted protein design tool to strategically add various glycosylation sites on the protein surface and create a panel of resurfaced stabilized core (RSC3)-based derivatives. The modified proteins were expressed in 293F or GnTi(-/-) cells and purified by Nickel-affinity and gel-filtration chromatography. To verify the glycosylation sites and antigenic analysis, the purified proteins were subject to mass spectrometry and SDS PAGE analysis using a panel of well-defined monoclonal antibodies. The proteins with desirable antigenicity and well-tolerated glycan additions were tested for their immunogenicity in mice and NHPs.

## Results

Two hyperglycosylated mutants containing five or six extra glycans (RS3.Y5 and RSC3. Y6.1) elicited CD4bs antibodies in significant quantities. Variant Y6.1 was further tested in the NHP model, in which Y6.1 elicited non-neutralizing CD4bs antibodies. In addition, a novel glycan mutant Y8.2_1 was developed based on Y6.1 by incorporating 3 extra glycans. Antigenic analysis indicated that the protein was able to eliminate the binding of non-neutralizing or less potent CD4bs antibodies such as b12 and b13, but still retain the binding capacities to potent CD4bs antibodies such as VRC01 and VRC-PG04.

## Conclusion

It is now feasible to induce CD4bs antibodies by vaccination with immunogens that have exogenous N-linked glycans added to mask the “undesired” regions of the immunogen and have an exposed surface that targets the CD4bs epitope. Further optimization will be necessary to obtain CD4bs antibodies with neutralizing activity.

